# Mechanism of Negative Emotions of the Elderly in Normalization Period of COVID-19: A Mediated Mediation Model

**DOI:** 10.3389/fpubh.2022.941958

**Published:** 2022-06-28

**Authors:** Kai Xu

**Affiliations:** School of Educational Science, Luoyang Normal University, Luoyang, China

**Keywords:** the elderly, epidemic attention, negative emotion, subjective economic status, health perception

## Abstract

To explore the mechanism of negative emotions of the elderly in normalization period of COVID-19. The self-rating Depression Anxiety Stress Scale, epidemic attention scale, subjective economic status scale and physical health perception scale were used to investigate 318 elderly people in 2021. There were significant differences in negative emotions among the elderly in Henan in China with different gender, education background, medical insurance and whether they contacted suspected cases (all *P* < 0.05), but there was no significant difference on religious belief (*P* > 0.05); Attention to epidemic information was positively correlated with negative emotion (*r* = 0.492, *P* < 0.01), and negatively correlated with subjective economic status (*r* = −0.138, *P* < 0.05); Negative emotions were negatively correlated with subjective economic status (*r* = −0.455, *P* < 0.01) and health perception (*r* = −0.277, *P* < 0.01); health perception was no significant correlation with epidemic attention(*r* = −0.047, *P* > 0.05) and subjective economic status (*r* = −0.033, *P* > 0.05). Bootstrap test found that epidemic attention can significantly predict negative emotion of the elderly (β = 0.492, *P* < 0.001), subjective economic status played a partial mediating role between epidemic attention and negative emotions (β = 0.438, −0.395, *P* < 0.001), and health perception moderated the first half of the mediating path (β = 0.403, *P* < 0.001, 95% CI = [0.286~0.521]). Epidemic attention has a significant positive impact on the negative emotions of the elderly in Henan during normalization period of COVID-19, and it has effect indirectly through subjective economic status; health perception plays a moderator role in the impact of epidemic attention on subjective economic status.

## Introduction

As a major public health emergency, COVID-19 is characterized by multiple transmission routes, long incubation period, strong infectivity and rapid spread. It is difficult to prevent and control the epidemic. It not only threatens people's life and physical health, but also brings various impacts on people's mental health. Due to the degenerative changes of various physical and mental functions, the elderly have become a vulnerable group in the society, which is prone to negative emotional experience, resulting in serious psychological problems (1). In this epidemic, due to the weak immune function, the elderly have become the susceptible group and high-risk vulnerable group of this infectious disease, and most of the elderly in the critically ill group. The study also found that during the epidemic period, the incidence of depression and anxiety in the elderly increased significantly (2), and they were more prone to poor mental health and anxiety symptoms (3), which was the focus of social psychological counseling and crisis intervention during the epidemic period. At present, there are few studies on the mental health of the elderly in Chinese normalization period of COVID-19, which mainly focusing on the current situation and influencing factors of the frailty of the elderly in the community and the health management strategy of the elderly with chronic diseases (4, 5). Therefore, how to understand the law of behavior and emotion of the elderly in time and guide them to respond to public health emergencies rationally and scientifically has become an urgent topic to be studied.

In major emergencies such as the epidemic, people's mental health problems are mainly manifested in three negative emotions: stress, depression and anxiety (6). At present, there have been some researches on the mental health of the elderly during the epidemic period, but there are still deficiencies in the influence mechanism of the negative emotion of the elderly in China during the epidemic period, which needs to be further clarified. The important factor affecting people's mental health in the early stage of the epidemic is the epidemic information (7), people's cognitive uncertainty exists for a long time (8), and they tend to process negative information, which will lead to various serious negative emotional problems (9). Therefore, this study puts forward hypothesis 1:

Epidemic concern has a significant positive impact on the negative emotions of the elderly.

Socio economic status is an important concept in the theory of social stratification. Socio economic status is the position of an individual in society and the comprehensive embodiment of his education level, occupation, income, residence and other indicators (10). The research on China's national mental health literacy found that in terms of social influencing factors, socio-economic status is the most effective factor among all variables, and its interpretation rate of Chinese mental health literacy is the highest, that is, the higher the socio-economic status, the higher the mental health literacy (11). Socio economic status is often divided into objective socio-economic status and subjective socio-economic status. The latter refers to the evaluation of their socio-economic status by the elderly compared with their peers. In the impact on the mental health of the elderly, the subjective economic status has an important impact. The higher the social class positioning, the better the mental health of the elderly (12). Therefore, this study puts forward hypothesis 2:

Subjective economic status has a significant negative impact on the negative emotion of the elderly, and plays an intermediary role between epidemic concern and negative emotion.

Physical health perception is an individual's subjective perception and evaluation of self-health. Physical health perception level is a good indicator of health status. It can not only reflect individual health status, but also predict mortality (13). In the face of all kinds of information about the COVID-19, the elderly are more sensitive than other people. Their bodies will release a large number of signals, resulting in autonomic nervous symptoms such as elevated blood pressure, panic, suffocation, asthma, sweating, and various physical discomfort (14). The sense of physical health is closely related to negative emotions. Individuals with lower physical health perception have more negative emotions, and individuals with better physical health perception have more positive emotions (15). It can be seen that under the same conditions of epidemic concern and subjective economic status, the elderly with low physical perception will have more negative emotions, and the elderly with high physical health perception will have less negative emotions. Therefore, this study puts forward hypothesis 3:

Physical health perception plays a regulatory role in the impact of epidemic concern on subjective economic status.

**Figure 1:**
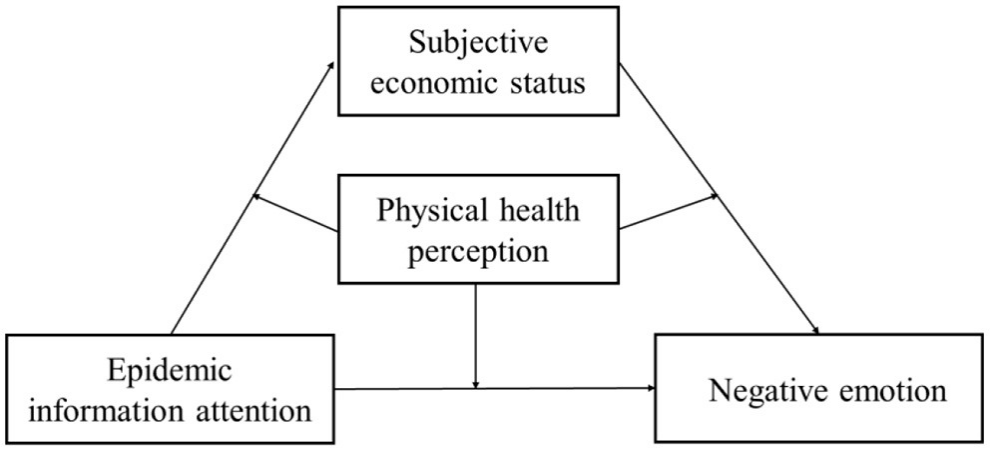
Hypothetical model diagram of this study.

To sum up, this study proposes a moderated mediation model for the impact mechanism of negative emotions of the elderly during the COVID-19 period, as shown in Figure 1. Specifically, it includes the intermediary role of subjective economic status between epidemic concern and negative emotion, as well as the regulatory role of physical health perception in the direct path and intermediary path, so as to provide scientific support and thinking for effective psychological counseling and assistance to the elderly in the period of epidemic normalization.

## Research Object and Method

### Research Object

Using the method of convenient sampling, 318 elderly people were investigated in Henan Province from March to May 2021. Inclusion criteria: (1) age ≥ 55 years old; (2) Be conscious and able to answer questions independently; (3) Informed consent to this questionnaire and voluntary acceptance of this study. Exclusion criteria: (1) those who refuse to investigate; (2) Those with cognitive and language barriers cannot accurately understand the questionnaire items; (3) People with serious physical and mental diseases. The questionnaire adopts unified guiding language, and the elderly in their families are investigated by Henan students from their departments and teaching departments. Since face-to-face investigation cannot be conducted during the epidemic, the online structured self-filling electronic questionnaire is adopted. After obtaining the informed consent of the elderly, the questionnaire is distributed by sharing the QR code and link of the questionnaire, which is filled in by the subjects themselves. The study passed the review of ethics of Luoyang Normal University.

### Research Tool

(1) Demographic characteristics questionnaire: The contents of the questionnaire include gender, age, educational background, religious belief, whether there is medical insurance, whether there is contact with suspected cases, etc.
(2) Measurement of negative emotions: The self-rating Depression Anxiety Stress Scale (DASS-21) was developed by Lovibond et al. (16). Anxiety, depression and stress are three typical negative emotional experiences, and they are also the research hotspots of mental health in theory and clinic. Accurate and rapid diagnosis and differentiation of individual negative emotional status is the prerequisite to ensure individual mental health (17). Therefore, this study uses DASS-21 to measure mental health. Studies have confirmed that DASS-21 has good reliability and validity in the Chinese elderly (18–20). There are 21 items in the scale, which is divided into three subscales: depression, anxiety and stress. Each subscale contains 7 items. This scale adopts a 5-level score to allow the subjects to judge the consistency between their recent negative emotional experience or corresponding physiological response and the description of each item. The higher the score, the lower the level of mental health (21, 22). Diagnostic criteria for DASS-21 scores are detailed in Table 1.
(3) This paper adopts the epidemic concern questionnaire (23), which includes a question item (“how much do you pay attention to the epidemic situation of coronavirus pneumonia recently?”). The questionnaire adopts 5-level scoring. The higher the score, the higher the degree of concern about the epidemic.
(4) Subjective economic status scale: We used the MacArthur scale of subjective SES (24, 25) to present a 5-step ladder to the subjects, which means that the economic income is getting higher and higher from bottom to top. The subjects were asked to self-evaluate that their family economic income was at the level of the ladder, so as to represent their perceived subjective economic status.
(5) Physical health perception scale: Based on the overall physical health perception (HPQ) scale of HEO et al. (26), our survey of the elderly in China has good reliability and validity (27). The scale includes 4 items, e.g., “How would you rate your own health at present?” and “What do you think of your own health condition compared to that of other men=women of your age?”. Using a five-point Likert scale, respondents were asked to answer from “bad” (1) to “excellent (5).” A high score indicated a better perception of health.

**Table 1:** Diagnostic criteria for DASS-21 scores.

	**Depression**	**Anxiety**	**Stress**
None	0–4	0–3	0–7
Mild	5–6	4–5	8–9
Medium	7–10	6–7	10–12
heavier	11–13	8–9	13–16
Serious	≥14	≥10	≥17

### Quality Control

In order to control the quality of questionnaire collection, each IP address limit is set in the background to answer once; In order to improve the authenticity of the questionnaire, the questionnaire does not involve personal information, and there is no reward, highlighting the initiative of the respondents; In order to control the validity of the questionnaire, each question item is set as a required item. If there are unanswered questions, they cannot be submitted, so as to avoid the existence of missing values.

### Statistical Method

SPSS 23.0 was used for descriptive statistics and correlation analysis, and the PROCESS 3.2 program developed by Hayes was used for moderated mediation test. The bootstrapping sampling times were 5000 and the test level was high α = 0.05.

## Results and Analysis

### Common Method Deviation Detection

Harman single factor test was used for common method deviation test (28). The results show that there are 6 factors with characteristic roots >1. The variation explained by the first factor is 26.63%, which is less than the critical standard of 40%, indicating that there is no serious common method deviation variation in this study (29).

### Comparison of Negative Emotion Levels in Different Demographic Characteristics

There are significant differences in the scores of negative emotions among the elderly with different gender, educational background, whether they have medical insurance or not and whether they are exposed to suspected cases, but there is no significant difference in age and whether they have religious beliefs, as shown in Table 2. In terms of gender, the male elderly were significantly higher than the female elderly (*P* < 0.001); In terms of age, there was no significant difference in negative emotion scores between 55 and 65 years old group and > 65 years old group; In terms of educational background, the negative emotion of the elderly with primary school education and below is the highest, and the post test (LSD) shows that it is significantly higher than that with high school education; In terms of medical insurance, the elderly without medical insurance have the highest negative emotion score, which is significantly higher than those with partial medical insurance and complete medical insurance; In terms of whether to contact suspected cases, the negative emotions of the elderly with suspected cases were significantly higher than those without contact (*P* < 0.01).

**Table 2:** Comparison of negative emotion scores of the elderly with different demographic characteristics.

**Project**	**Number** **of people**	**Negative** **emotion**	***T/F***	***P***
Male	179	52.48 ± 13.68	4.897	<0.001
Female	139	44.79 ± 14.15		
55–65 age	201	50.20 ± 14.72	1.768	0.078
>65 age	117	47.26 ± 13.66		
Primary school education and below	28	56.14 ± 14.50	3.017	0.030
Junior high school education	28	49.11 ± 13.08		
High school education (including technical secondary school)	217	47.88 ± 14.53		
University degree or above	45	50.76 ± 13.36		
No religion	288	49.44 ± 14.39	1.236	0.217
Have religion	30	46.03 ± 14.20		
No health insurance	6	69.00 ± 5.87	6.983	0.001
Some medical insurance	102	50.29 ± 14.51		
Fully medicare	210	47.98 ± 14.07		
Contact with suspected cases	8	60.75 ± 8.83	3.698	0.006
Non-contact with suspected cases	310	48.82 ± 14.38		

### Descriptive Statistical Analysis and Correlation Analysis

The mean value, standard deviation and correlation matrix of each variable are shown in Table 3. Epidemic concern was significantly positively correlated with negative emotion and negatively correlated with subjective economic status; Negative emotion was negatively correlated with subjective economic status and physical health perception; There was no significant correlation between physical health perception and epidemic concern and subjective economic status. According to the establishment criteria of intermediary variables and regulatory variables proposed by Wen et al., if a variable has little correlation with independent variables or dependent variables, it cannot become an intermediary variable, but it can become a better regulatory variable (30). It can be seen that subjective economic status is significantly negatively correlated with epidemic concern and negative emotion, which can be used as an intermediary variable; However, there is no significant correlation between physical health perception and epidemic concern, which is a better regulatory variable.

**Table 3:** Correlation coefficients between variables.

	***M***	***SD***	**1**	**2**	**3**	**4**
Epidemic concern	4.62	0.52	—			
Negative emotions	49.12	14.38	0.492^**^	—		
Subjective economic status	3.71	0.85	−0.138^*^	−0.455^**^	—	
Perception of physical health	15.81	3.45	−0.047	−0.277^**^	−0.033	—

*N = 318;*

* *p < 0.05;*

** *p < 0.01;*

^***^p < 0.001.

### Moderated Mediation Model Test

According to Wen et al. (31, 32), a moderated mediation model needs to meet the following four conditions: (1) The effect of independent variables on dependent variables is significant; (2) The effect of independent variables on mediator variable is significant; (3) The effect of intermediary variables on dependent variables is significant; (4) The interaction between independent variables and moderator variable has significant effect on mediator variable, or the interaction between mediator variable and moderator variable has significant effect on dependent variables. Therefore, this article needs to test: (1) epidemic concern has a significant effect on negative emotions; (2) Epidemic concern has a significant effect on subjective economic status; (3) Subjective economic status has a significant effect on negative emotion; (4) The interaction between epidemic concern and physical health perception has a significant effect on subjective economic status, or the interaction between subjective economic status and physical health perception has a significant effect on negative emotion. The first three conditions are the test of the mediating effect of subjective economic status between epidemic concern and negative emotion, and the fourth condition is the test of the moderating effect of physical health perception on this mediating model. Before the test, according to the standardization requirements of mediation and moderating effects, this study centralizes all variables, and then multiplies the scores of physical health perception and epidemic concern, as well as the scores of physical health perception and subjective economic status as interactive items (33).

(1) Intermediary model test of subjective economic status

The first three conditions were tested by multiple stepwise regression analysis, that is, to verify the intermediary role of subjective economic status between epidemic concern and negative emotion. The results showed that in the first step regression equation, epidemic concern had a significant positive predictive effect on the negative emotions of the elderly (β = 0.492, *t* = 10.058, *P* < 0.001), indicating that epidemic concern can promote negative emotion; In the second step regression equation, epidemic concern has a significant negative predictive effect on subjective economic status (β = −0.138, *t* = −2.479, *P* < 0.05); The third step is to incorporate the independent variable epidemic concern and the intermediary variable subjective economic status into the regression equation. It is found that the subjective economic status has a significant negative predictive effect on the negative emotion of the elderly (β = −0.395, *t* = −8.917, *P* < 0.001), indicating that subjective economic status can delay negative emotions, and emotional information attention can still significantly predict negative emotions (β = 0.438, *t* = 9.899, *P* < 0.001), indicating that subjective economic status plays a partial mediating role between epidemic concern and negative emotion. As shown in Table 4, the first three conditions have been met.

**Table 4:** Intermediary model test of subjective economic status.

**Regression equation**		**Overall fitting index**				**Significance of regression coefficient**	
**Result variable**	**Predictive variable**	***R***	***R*** ^**2**^	***SE***	***F***	**β**	***t***
Negative emotion	Epidemic concern	0.492	0.242	0.872	101.160^***^	0.492	10.058^***^
Subjective economic status	Epidemic concern	0.138	0.019	0.992	6.146^*^	−0.138	−2.479^*^
Negative emotion	Epidemic concern	0.629	0.395	0.780	102.900^***^	0.438	9.899^***^
	Subjective economic status					−0.395	−8.917^***^

*N = 318;*

* *p < 0.05;*

^**^p < 0.01;

*** *p < 0.001*.

(2) The moderator role of physical health perception in mediating model

The fourth condition uses model 59 in the process program developed by Hayes to test the moderated mediation model, and takes physical health perception as the regulating variable into the equation. The results are shown in Table 5. Epidemic concern and physical health perception have significant negative prediction on subjective economic status, and their interaction items have significant positive prediction on subjective economic status (β = 0.403, *P* < 0.001), indicating that physical health perception has a moderating effect on epidemic concern → subjective economic status; Epidemic concern and physical health perception have significant negative predictive effects on negative emotions, but the interaction between epidemic concern and physical health perception and the interaction between subjective economic status and physical health perception have no significant predictive effects on negative emotions (β _Interactionitem__1_ = −0.099, *P*> 0.05; β _Interactionitem2_ = 0.055, *P* > 0.05), indicating that physical health perception has no moderating effect on epidemic concern → negative emotion, subjective economic status → negative emotion. It can be seen that physical health perception plays a moderator role in the first half path of epidemic concern → subjective economic status → negative emotion mediation model, but does not play a regulatory role in the second half path of mediation model and in the direct path of epidemic concern to negative emotion.

**Table 5:** Moderated mediation model test.

**Result variable**	**Predictive variable**	***R***	***R*** ^**2**^	***SE***	***F***	**β**	***t***	**95% CI**
Subjective economic status		0.381	0.145	0.863	17.725^***^			
	Epidemic concern					−0.154	−2.954^**^	[−0.257 to −0.052]
	Physical health perception					−0.103	−1.944	[−0.208~0.001]
	Epidemic concern × Physical health perception					0.403	6.752^***^	[0.286~0.521]
Negative emotion		0.692	0.479	0.530	57.323^***^			
	Epidemic concern					0.416	9.585^***^	[0.331~0.502]
	Subjective economic status					−0.369	−8.350^***^	[−0.456 to −0.282]
	Physical health perception					−0.248	−5.914^***^	[−0.330 to −0.165]
	Epidemic concern × physical health perception					−0.099	−1.946	[−0.199~0.001]
	Subjective economic status × physical health perception					0.055	1.287	[−0.029~0.139]

*N = 318;*

^*^p < 0.05;

** *p < 0.01;*

*** *p < 0.001*.

**Figure 2:**
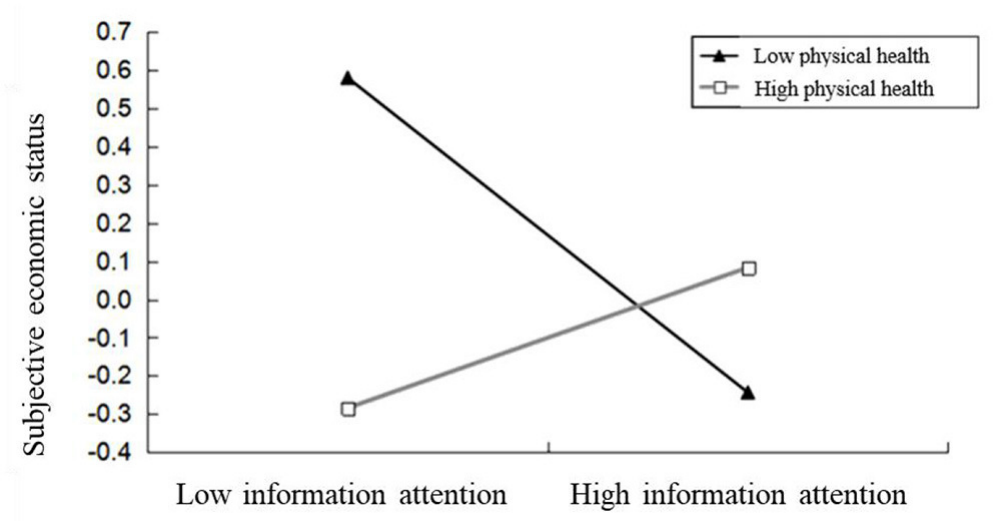
The moderator role of physical health perception between epidemic concern and subjective economic status.

In order to specifically explore the moderating effect of physical health perception, add or subtract a standard deviation from the average value of physical health perception and epidemic concern score, and divide it into low physical health perception group, high physical health perception group, low epidemic concern group and high epidemic concern group. Make the slope diagram of moderating effect (as shown in Figure 2) to further analyze its simple effect. The results showed that in the group with low level of physical health perception, epidemic concern significantly negatively predicted subjective economic status (*t* = −6.888, *P* < 0.001); In the group with high level of physical health perception, epidemic concern significantly positively predicted subjective economic status (*t* = 3.201, *P* < 0.01). The results show that physical health perception plays a regulatory role between epidemic concern and subjective economic status.

## Discussion

This study found of the elderly in the COVID-19 period were very serious, the number of elderly people without depression, anxiety or stress in the COVID-19 is 0, and most of them are in serious and severe degrees, with anxiety being the most serious, followed by stress and depression, shown in Table 6. 44.03% of the elderly were in a state of serious depression, 94.34% of the elderly were in a state of serious anxiety, and 70.13% of the elderly were in a state of serious stress. It can be seen that the elderly in the epidemic period, as a vulnerable group, are a group that needs to be cared for in social psychological counseling and crisis intervention.

**Table 6:** Number and proportion of people with different symptoms of depression, anxiety and stress.

	**Depression**	**Anxiety**	**Stress**
None	0	0	0
Mild	40 (12.58%)	0	4 (1.26%)
Medium	61 (19.18%)	4 (1.26%)	40 (12.58%)
heavier	77 (24.21%)	14 (4.40%)	51 (16.04%)
Serious	140 (44.03%)	300 (94.34%)	223 (70.13%)

Like the conclusions of other studies, the anxiety, depression and stress of the elderly men are significantly higher than those of women (2). The elderly in different age groups do not score significantly in negative emotions, because COVID-19 is a high-intensity stressful life event, which poses a major threat to the mental health of all people, and has become an important stressor causing people's psychological balance disorder (34). The elderly with primary school education and below have the highest negative emotions, which may be that the teachers with low education level lack effective information about the epidemic and lack confidence in how to prevent the epidemic, resulting in a large degree of anxiety, stress and depression. Previous studies have found that religious belief has a significant positive impact on the health status of the elderly, and its important way is to participate in religious activities. This social capital helps to alleviate the loneliness of elderly believers (35). This study found that religious beliefs have no significant impact on the negative emotions of the elderly under the epidemic. The reason for this phenomenon is that for the sake of epidemic prevention and control, gathering activities are limited, and religious worship and other activities are also greatly affected. To some extent, this finding shows that the mental health of Chinese elderly believers mainly benefits from external religious orientation rather than internal religious orientation. Different from the research of Zhou Qin and others, old-age insurance such as the new rural cooperative medical insurance, as a stable source of income, has played a positive role in improving residents' mental health, while the corresponding role of medical insurance systems such as the new rural cooperative medical system is basically not significant (36). This study found that medical insurance can greatly improve the mental health of the elderly, which is related to the special period of the epidemic. The elderly exposed to suspected cases have significant negative emotions because they are worried about being infected and are transmitted to their families.

Correlation analysis showed that epidemic concern was significantly positively correlated with negative emotion and negatively correlated with subjective economic status; Negative emotions are significantly negatively correlated with subjective economic status and physical health perception. Further analysis shows that epidemic concern has a significant positive predictive effect on negative emotions. This conclusion is consistent with the previous research results (3). The more concerned about epidemic information, the more sensitive to various information, which is more likely to lead to certain negative emotions.

The mediating effect model shows that subjective economic status plays a partial mediating role between epidemic concern and negative emotion, indicating that epidemic concern directly leads to the generation of negative emotional experience of the elderly on the one hand, and indirectly reduces the generation of negative emotional experience of the elderly through subjective economic status on the other hand. This shows that economic status is an important factor affecting the physical and mental health of the elderly in Normalization Period of COVID-19. Existing studies have also confirmed that Socio economic status is one of the fundamental factors affecting people's physical and mental health (37); the improvement of economic level will help individuals obtain better positive psychological resources, which will directly improve the mental health of the elderly and reduce the occurrence of emotional and behavioral problems (38).

The interaction between epidemic concern and physical health perception has a significant positive prediction on subjective economic status, indicating that physical health perception plays a moderating effect in the impact of epidemic concern on subjective economic status. Further simple slope analysis shows that different levels of physical health perception play different roles in the relationship between epidemic concern and subjective economic status: the older people with low-level physical health perception pay more attention to the epidemic, the lower their subjective economic status, and the lower their attention to the epidemic, the higher their subjective economic status; Different from the elderly with high-level physical health perception, the lower their attention to the epidemic, the lower their subjective economic status. The more they pay attention to the epidemic, the higher their subjective economic status. According to the theory of limited cognitive resources, people's cognitive resources are limited, which makes people have to allocate limited resources to different activities or different aspects of the same activity (39). When the elderly are weak and ill, they will continue to pay attention to their health, so that their cognitive resources have been occupied. Therefore, those who allocate more cognitive resources to obtain epidemic information are often those with poor economic conditions. Compared with those with good economic conditions, they are more likely to perceive the threat of the epidemic to their health; Correspondingly, the cognitive resources of healthy elderly people do not focus too much on their own physical health. They pay more attention to the external world. The more types of activities they participate in, the better their health will benefit. These are closely related to economic conditions (40). Therefore, those who pay more attention to epidemic information are often those with better economic conditions.

## Conclusion

To sum up, this study found that subjective economic status and physical health perception play a regulatory intermediary role between epidemic concern and negative emotions, which is of great significance to prevent and reduce the occurrence of psychological crisis and effectively carry out psychological counseling for the elderly during the epidemic period. Firstly, the intermediary role of subjective economic status suggests that we can start from the aspects of medical insurance, ensuring the living standard of low-income people and increasing income; Secondly, the differential moderating effect of physical health perception suggests that different targeted strategies should be adopted in prevention and intervention. For the elderly with low physical health, we should focus on life security and physical health maintenance, while for the elderly with good physical health, we should release effective epidemic information to guide them to actively participate in epidemic prevention and control.

## Data Availability Statement

The original contributions presented in the study are included in the article/supplementary material, further inquiries can be directed to the corresponding author/s.

## Author Contributions

KX: research, data analysis, writing, and revising papers.

## Funding

This work was supported by Henan Key R&D and Promotion Special Project (soft science research) (212400410306); Henan Philosophy and Social Science Planning Project (2021BZZ007); National Natural Science Foundation of China (72074235); Henan Research and Practice Project of Higher Education Teaching Reform (2021SJGLX496).

## Conflict of Interest

The author declares that the research was conducted in the absence of any commercial or financial relationships that could be construed as a potential conflict of interest.

## Publisher's Note

All claims expressed in this article are solely those of the authors and do not necessarily represent those of their affiliated organizations, or those of the publisher, the editors and the reviewers. Any product that may be evaluated in this article, or claim that may be made by its manufacturer, is not guaranteed or endorsed by the publisher.

## References

[1] Liu HL Tian QH. Ways to improve the mental health level of the elderly from the perspective of community activities. Chin J Gerontol. (2019) 14:3571–6. 10.3969/j.issn.1005-9202.2019.14.067

[2] Chen XY Chen Z Ma JL. Study on the psychological status and influencing factors in the elderly living in nursing home under long-term fully closed management during the epidemic period of COVID-19 in low risk area. Practical Geriatrics. (2020) 12:1329–32. 10.3969/j.issn.1003-9198.2020.12.033

[3] Gao R Zhang BZ Peng SR. Mental health status of the general population during the COVID-19 epidemic in Ya'an City. Chin Mental Health J. (2021) 1:79–84. 10.3969/j.issn.1000-6729.2021.01.014

[4] Wang YL Chen H Chen HJ. Research on the status quo and influencing factors of frailty among the elderly in the community. Chin Clin Nurs. (2022) 4:203–6. 10.3969/j.issn.1674-3768.2022.04.002

[5] Yang ZK Shi XN Yang XT. Discussion on the health management mode of the elderly with chronic diseases under the background of the normalization of epidemic prevention and control. Chin J Public Health Manage. (2021) 5:616–9. 10.19568/j.cnki.23-1318.2021.05.0013

[6] Hardy GE Woods D Wall TD. The impact of psychological distress on absence from work. J Appl Psychol. (2003) 2:306–14. 10.1037/0021-9010.88.2.306 12731714

[7] Wang JX Ying XP. Cognition, emotion and action: social mentality in the emergency response. Exploration Free Views. (2020) 4:232–43.

[8] Brashers DE. Communication and uncertainty management. J Commun. (2001) 3:477–97. 10.1111/j.1460-2466.2001.tb02892.x

[9] Sprengelmeyer R Jentzsch I. Event related potentials and the perception of intensity in facial expressions. Neuropsychologia. (2006) 14:2899–906. 10.1016/j.neuropsychologia.2006.06.020 16959277

[10] Maeland JG. Socioeconomic inequalities in health. Norsk Epidemiologi. (2009) 1:557–66. 10.5324/nje.v12i1.498

[11] Jiang GR Li DY Ren ZH Yupeng Y Xinchun W Xu Z. The status quo and characteristics of Chinese mental health literacy. Acta Psychol Sinica. (2021) 2:182–201. 10.3724/SP.J.1041.2021.00182

[12] Wang XH. The impact of socio-economic status on the subjective well-bei g of the elderly. J Dalian University Technol. (2021) 3:92–100. 10.19525/j.issn1008-407x.2021.03.011

[13] Pu C Tang GJ Huang N Chou YQ. Predictive power of self-rated health for subsequent mortality risk during old age: analysis of data from a nationally representative survey of elderly adults in Taiwan. J Epidemiol. (2011) 4:278–84. 10.2188/jea.JE20100131 21606607 PMC3899420

[14] Pu CC Wang HL. Psychological reactions and coping strategies of the elderly facing COVID-19. Chin J Mental Health. (2020) 3:257–8. 10.3969/j.issn.1000-6729.2020.3.024

[15] Pressman SD Cohen S. Does positive affect influence health? Psychol Bull. (2005) 6:925. 10.1037/0033-2909.131.6.925 16351329

[16] Lovibond P F Lovibond S H. The structure of negative emotional states: Comparison of the Depression Anxiety Stress Scales (DASS) with the Beck Depression and Anxiety Inventories. Behav Res Ther. (1995) 3:335–43. 10.1016/0005-7967(94)00075-U 7726811

[17] Li XL Tang HB Guo F. A review of depression-anxiety-stress scale's reliability and validity. Chin J Clin Psychol. (2012) 3:350–2. 10.16128/j.cnki.1005-3611.2012.03.039

[18] Gong X Xie XY Xu R Luo Y. Psychometric properties of the Chinese versions of DASS-21 in Chinese college students. Chin J Clin Psychol. (2010) 4:443–6.

[19] Wen Y Wu DX Lv XJ Li H. Psychometric properties of the Chinese short version of depression anxiety and stress scale in Chinese adults. Chin J Public Health. (2012) 11:1436–8. 10.11847/zgggws2012-28-11-14

[20] Sun Y Yan F Wang WT. Study on the moderated effect of migration years on the relationship between stress and depression and anxiety among the elderly in new cities. General Pract China. (2017) 2:210–3. 10.3969/j.issn.1007-9572.2017.02.018

[21] Crawford JR Henry JD. The Depression Anxiety Stress Scales (DASS): Normative data and latent structure in a large non-clinical sample. Br J Clin Psychol. (2003) 2:111–31. 10.1348/014466503321903544 12828802

[22] Lovibond SH Lovibond PF. Manual for the Depression anxiety Stress Scales. Sydney, NSW: Psychology Foundation. (1995). 10.1037/t01004-000

[23] Wang JX Gao WJ Chen MQ. Investigation report on social psychology under the COVID-19. Governance. (2020) 1:55–64. 10.16619/j.cnki.cn10-1264/d.2020.z1.014

[24] Adler NE Epel ES Castellazzo G Ickovics JR. Relationship of subjective and objective social status with psychological and physiological functioning: Preliminary data in healthy, White women. Health Psychol. (2000) 6:586–92. 10.1037/0278-6133.19.6.586 11129362

[25] Shang SJ Bai BY Zhong N. Family social class and meaning in life: mediating of basic psychological need satisfaction. Chin J Clin Psychol. (2016) 6:1108–11. 10.16128/j.cnki.1005-3611.2016.06.032

[26] Heo J Lee Y. Serious leisure, health perception, dispositional optimism, and life satisfaction among senior games participants. Educ Gerontol. (2010) 2:112–26. 10.1080/03601270903058523

[27] Liu F. Relationship Between Health Perception and Death Anxiety and Its Mechanism of Old People. Sichuan: Sichuan Normal University. (2015).

[28] Zhou H Long LR. Statistical remedies for common method biases. Adv Psychol Sci. (2004) 6:942–50.

[29] Xiong HX Zhang J Ye BJ. Common method variance effects and the models of statistical approaches for controlling it. Adv Psychol Sci. (2012) 5:757–69. 10.3724/SP.J.1042.2012.00757

[30] Wen ZL Hou JT Zhang L. A comparison of moderator and mediator and their applications. Acta Psychol Sinica. (2005) 2:268–74. 10.1111/j.1744-7909.2005.00136.x

[31] Wen ZL Zhang L Hou JT. Mediated moderator and moderated mediator. Acta Psychol Sinica. (2006) 3:448–52. 10.1016/S0379-4172(06)60092-9 16875320

[32] Wen ZL Liu HY Hou JT. Analysis of Moderating and Mediating Effect. Beijing: Educational Science Press. (2012) 91–2.

[33] Wen ZL Hou JT Marsh HW. Appropriate standardized estimates for moderating effects in structural equation models. Acta Psychol Sinica. (2008) 6:729–36. 10.3724/SP.J.1041.2008.00729

[34] Huang C Wang Y Wang Y Ren L Zhao J Hu Y. Clinical features of patients infected with 2019 novel corona virus in Wuhan, China. Lancet. (2020) 10:497–506. 10.1016/S0140-6736(20)30183-5 31986264 PMC7159299

[35] Jiang QC Zhang KZ. Does religious belief affect the health of the elderly? World Econ Papers. (2013) 5:85–106.

[36] Zhou Q Jiang WG Guo X. The effect of social insurance on mental health among rural residence: an empirical analysis based on CHARLS data. China Econ Stud. (2018) 5:125–36. 10.19365/j.issn1000-4181.2018.05.10

[37] Hu JR. The relationship between socio-economic status and physical function and health of the elderly – taking exercise and medical insurance as intermediary variables. J Beijing University Sci Technol. (2019) 4:61–9. 10.3969/j.issn.1008-2689.2019.04.009

[38] Lei XY Sun XT Strauss J Zhang P Zhao Y. Depressive symptoms and SES among the mid-aged and elderly in China: Evidence from the China Health and Retirement Longitudinal Study national baseline. Soc Sci Med. (2014) 11:224–32. 10.1016/j.socscimed.2014.09.028 25261616 PMC4337774

[39] Kahneman D Treisman A. Changing views of attention and automaticity. In: Parasuraman R Davis DR. Varieties of Attention. Orlando: Academic Press (1984) 29–61.

[40] Ding ZH. The influence of social participation of the rural oldest-old on their health. Lanzhou Acad J. (2018) 12:179–95. 10.3969/j.issn.1005-3492.2018.12.019

